# Changes in the Peripheral Blood Treg Cell Proportion in Hepatocellular Carcinoma Patients After Transarterial Chemoembolization With Microparticles

**DOI:** 10.3389/fimmu.2021.624789

**Published:** 2021-02-24

**Authors:** Zhizhong Ren, Yuanxun Yue, Yuewei Zhang, Jiahong Dong, Ying Liu, Xiaowei Yang, Xin Lin, Xueqiang Zhao, Zhanqi Wei, Yu Zheng, Tianxiao Wang

**Affiliations:** ^1^ Hepatobiliary Pancreatic Center Department, Beijing Tsinghua Changgung Hospital Affiliated to Tsinghua University, Beijing, China; ^2^ Department of Interventional and Pain, Beijing Luhe Hospital, Capital Medical University, Beijing, China; ^3^ School for Medicine, Institute for Immunology, Tsinghua University, Beijing, China

**Keywords:** hepatocellular carcinoma, Treg cells, m-TACE, tumor immunity, flow cytometry

## Abstract

**Objective:**

Transarterial chemoembolization (TACE) stands for an ideal therapy for patients with intermediate stage HCC. This study was carried out to observe the effect of microparticles-transarterial chemoembolization (microparticles-TACE, m-TACE) on the immune function of hepatocellular carcinoma (HCC) patients by detecting the proportion of regulatory (Treg) cells in the peripheral blood of HCC patients before and after m-TACE, and to determine whether m-TACE has a positive regulatory effect on the immune function of HCC patients.

**Methods:**

33 HCC patients treated with Gelatn Sponge Microparticles (GSMs-TACE) were enrolled. Flow cytometry was used to determine the proportion of Treg cells and CD4+/CD8+ T cells in peripheral blood of HCC patients 1 day before GSMs-TACE, 1 to 2 weeks and 3 to 5 weeks after GSMs-TACE, respectively.

**Results:**

The Tregs cell proportion of HCC patients was significantly higher than that of the healthy and cirrhosis controls and was associated with various clinical indicators of HCC patients. The Treg cell proportion in HCC patients with BCLC stage C was higher than that of stage B patients; The Treg cell proportion at 1 to 2 weeks postoperatively was 8.54 ± 1.27%, which was significantly lower than that before the GSMs-TACE. The Treg cell proportion at 3 to 5 weeks postoperatively was 7.59 ± 1.27%, which continued to decline. The ratio of CD4+/CD8+ T cells was 1.31 ± 0.56, 1.86 ± 0.73, 1.76 ± 0.58% (P<0.01) respectively.

**Conclusion:**

These results indicated that m-TACE could exert a positive regulatory effect on the anticancer immune function of HCC patients, which may be used in combination with immune adjuvant therapies to enhance the efficacy of HCC.

## Introduction

Hepatocellular carcinoma (HCC) is the most common malignancy worldwide. Currently, liver transplantation and surgical resection remain the primary choice for HCC, but the overall survival of HCC patients is still depressing mainly due to local recurrence, distant metastasis, treatment resistance, and the lack of early diagnosis (1–3). TACE is one of the first-line treatment choices for HCC patients with specific clinical characteristics, such as stage B according to Barcelona Clinic Liver Cancer (BCLC) (4). The transcatheter delivery of both chemotherapeutic agents and embolizing agents contributes to a dual effect of cytotoxicity and ischemia in tumor tissues (4). Currently, there are many TACE modalities in clinical practice, such as conventional TACE (c-TACE), m-TACE and Doxorubicin Eluting Beads TACE (DEB-TACE) (4).

As the representative of absorbable particles, Gelatn Sponge Microparticles(GSMs) are characterized by chemical cross-linking and physical adsorption since they can be absorbed 7–15 days after arterial embolization. Since 2009, satisfactory efficacy and safety of GSMs-TACE in the treatment of HCC patients at stage B and C (BCLC classification) with a diameter of 150–350 μm/350–560 μm GSMs have been achieved in our team (5, 6). During GSMs-TACE procedures, under the premise of good liver function, the standard of terminating embolization is that the tumor blood supply artery is completely embolized. Compared with c-TACE, the application of TACE combined with different diameters microparticles in the treatment of HCC can lead to more significant tumor necrosis, especially the huge HCC.

Since the identification of tumor antigens, various approaches manipulating the immune system have been developed for cancer therapies (7). However, not all patients respond to immunotherapies, and one of the major obstacles is the formation of immunosuppressive tumor microenvironment which is filled with immunosuppressive cells such as Treg cells (8). Treg cells are a T-lymphocyte subset and help maintain immune homeostasis by controlling abnormal/excessive immune responses. Studies have found that Treg cells are also involved in development and progression of tumors *via* acting as s suppressor of effective antitumor immunity (7, 9). High infiltration by Treg cells into TME was observed in various types of tumors including HCC and was found to be correlated with poor prognosis (8–10). Therefore, strategies to reduce Treg cells and control the functions of Treg cells would be potentially effective anticancer therapies.

Many studies have confirmed the efficacy and safety of TACE procedures in treating HCC, and it was considered that the effects were mainly attributed to the blood supply blockade and the cytotoxic effects (11, 12). Except for the tumor necrosis, it was also observed that extrahepatic metastasis were reduced or even disappeared after GSMs-TACE in our clinical practice. Considering the close relationship between tumor metastasis and the immunosuppressive TME, these findings prompted us wonder if the effect of m-TACE was also partly lead to tumor necrosis, which promoted the positive regulatory effects on the anticancer immunity. However, there have been no related studies to testify this hypothesis. Thus, this study aimed to determine the changes in immune function indicated by the peripheral blood Treg cell proportion in HCC patients after m-TACE. The results of this study provided a piece of preliminary evidence that m-TACE may be used in combination with immune adjuvant therapies to increase the efficacy of HCC treatment.

## Patients and Methods

### Patients

**Figure 1:**
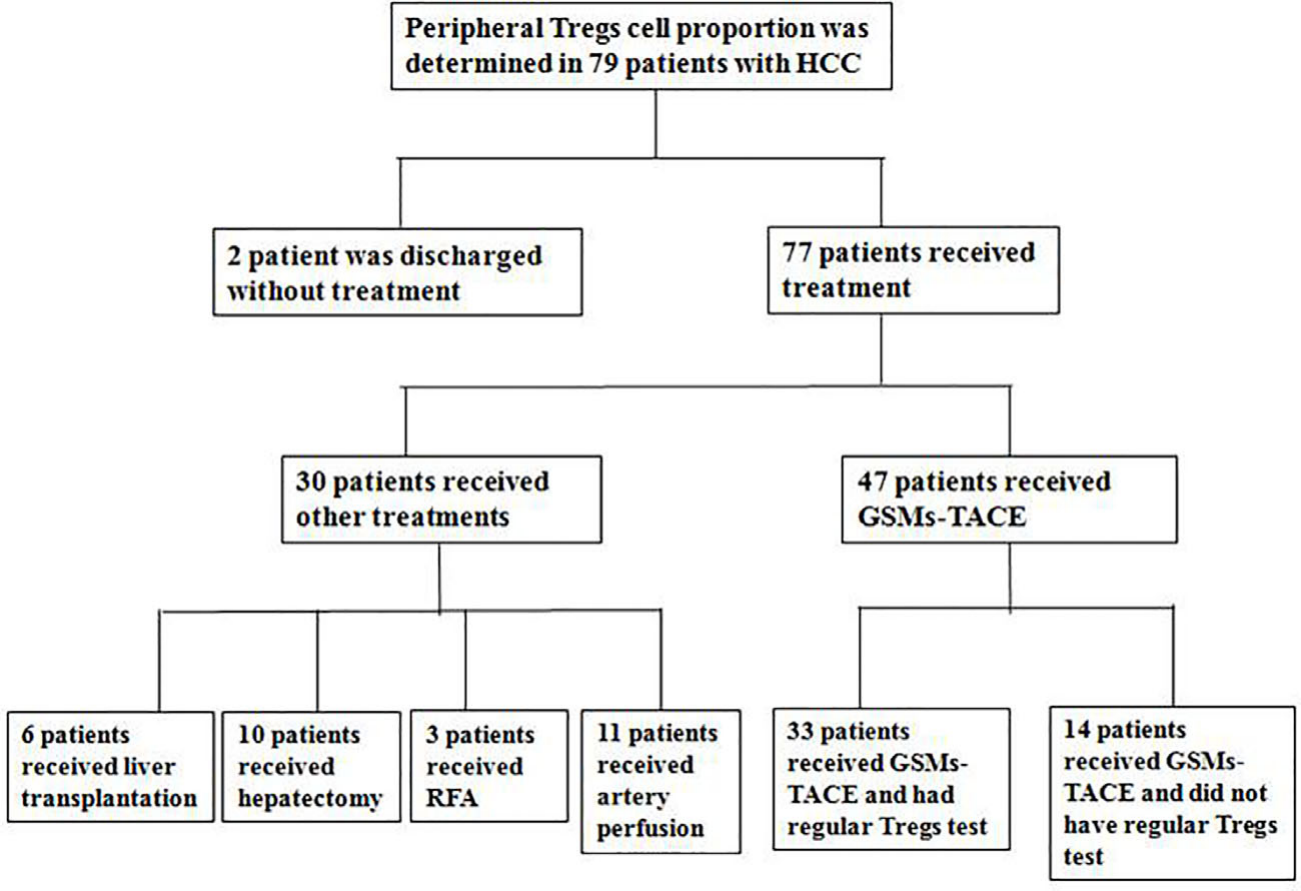
Flow chart of the enrollment of 33 HCC patients treated with GSMs-TACE. (RFA, radiofrequency ablation; HCC, hepatocellular carcinoma; GSMs-TACE, transarterial chemoembolization with gelatin sponge microparticles).

Seventy-nine patients with HCC diagnosed in our hospital from July 2017 to June 2020 were enrolled (**Figure 1**, **Table 1**). All patients were confirmed as HCC by two or more imaging examinations or liver tumor biopsy, according to the Expert Consensus on Regulation of Standardized Diagnosis and Treatment of Primary Liver Cancer. Thirty-three patients were followed up regularly after GSMs -TACE treatment for 3 to 5 weeks and the Treg cells proportion in peripheral blood was examined. Twenty healthy volunteers and 20 cirrhosis were selected as control group.

**Table 1:** Characteristics of 79 patients with HCC and 33 patients treated by GSMs-TACE who had regular Tregs test.

Variables	79 patient	33 patient
**Average age (years, mean ± SD)**	55 ± 12	54 ± 12
**Gender**		
Male	65	26
Female	14	7
**Child-Pugh grade**		
A	67	29
B	12	4
**BCLC stage**		
B	31	13
C	48	20
**AFP (ng/mL)**		
<400	32	17
≥400	47	16
**PIVKA-II(mAU/ml)**		
<1000	35	12
≥1000	44	21
**Tumor size (cm)**		
<10	41	12
≥10	38	21
**Tumor number**		
<4	43	14
≥4	36	19
**Extrahepatic metastasis (cases)**		
Yes	33	12
no	46	21
**Portal or hepatic vein invasion (cases)**		
Yes	42	19
no	37	14
**Tumor encapsulation**		
Yes	37	15
no	42	18
**Type of hepatitis**		
Hepatitis B	67	28
No hepatitis	12	5
**HBeAg**		
Positive	18	9
Negative	49	24

AFP, alpha-fetoprotein; PIVKA-II, Protein Induced by Vitamin K Absence or Antagonist-II; BCLC, Barcelona Clinic Liver Cancer (BCLC) staging system.

### The Standardized GSMs-TACE

The enrolled patients were treated with GSMs-TACE, all the blood supply arteries of the tumors were identified according to the location, size and staining integrity of the tumors. Microparticles of different sizes (150μm, 350–560 μm, and 560–710 μm) were selected during the procedure according to the tumor size and degree of staining. The appropriate dose of epirubicin (30–50 mg) was decided according to the tumor volume, and the dose of GSMs embolic agent (50–200 mg) was selected according to the tumor volume and degree of staining. Epirubicin was diluted with 50–100 mL of saline and then mixed with embolic agents of GSMs evenly. When the GSMs achieved a uniform and sparse suspension in epirubicin dilution, the mixture was used to slowly embolize the feeding artery of the tumor until the tumor staining disappeared completely, and the embolization usually lasted for 20–30 min.

### Collection and Process of Blood Sample

All patients signed the informed consent. Functions of liver and kidney, blood test and tumor marker (AFP and PIVKA-II) were routinely examined 1 day before GSMs-TACE and 4, 10, and 30 days after TACE. Upper abdominal CT plain scan was performed 4 days after operation, and enhanced CT or MRI was performed 30 days after TACE. Two milliliter forearm venous blood was collected from all patients with HCC 1 day before GSMs-TACE, 1 to 2 weeks and 3 to 5 weeks after TACE using blood collection tube treated with heparin sodium anticoagulant. Flow cytometry was used to determine Treg, CD4+T, CD8+T and NK cell proportions in peripheral blood. Two milliliter of forearm venous blood was also collected from the healthy and cirrhosis control group, and the data were collected for statistical analysis.

### Blood Preparation and Determination of Treg Cell Proportion by Flow Cytometry

1. Venipuncture blood was collected using anticoagulant tube;
2. One hundred milliliter whole blood was added to the bottom of the dry powder reagent tube which has antibodies of CD25/CD4/CD127/CD3 in the bottom;
3. the tube was vortexed for 0.5-1 s to mix well and incubated for 15 min at room temperature with light avoided;
4. Five hundred microliter of erythrocyte lysate was added to the tube. The tube was then incubated for 15 min at room temperature with light avoided;
5. Two milliliter PBS solution was added to each tube, and then oscillate the tube. Then the mixture was centrifuged at room temperature for 5 min with a centrifugal force of 300 g;
6. the supernatant was removed, and then step 5 was repeated for one more time.
7. the supernatant was removed, and then 500 mL PBS solution was added to each tube. The sample was then used for flow cytometry within 1 h;
8. the gating strategy of flow cytometry was set as follows: Treg: cell population of ①CD4^+^ and CD3^+^; ②CD25^+^ and CD127^+^ low.

### Flow Cytometry for CD4+T CD8+T in Peripheral Blood

The cell surface expression levels of CD4, CD8 were evaluated using flow cytometry, followed by incubation with PE-CY5-conjugated anti-CD4 antibody and FITC-conjugated anti-CD8 antibody at room temperature. After 10 min, red cells were removed using lysis buffer, and were washed twice with PBS. The cells were resuspended in 0.5 ml PBS. Analysis was performed on the results obtained from at least 10,000 cells, which were acquired on a CytoFLEX (Beckman Coulter).

### Statistical Analysis

The statistical analysis was conducted by SPSS software (provided by IBM, version 20.0). Results were expressed as mean ± SD. The *t* test was used to compare the data of different groups. The test level was α=0.05, and P < 0.05 was considered statistically significant.

## Results

### The Increased Proportion of Treg Cells in Peripheral Blood of Cirrhosis and HCC Patients

**Figure 2:**
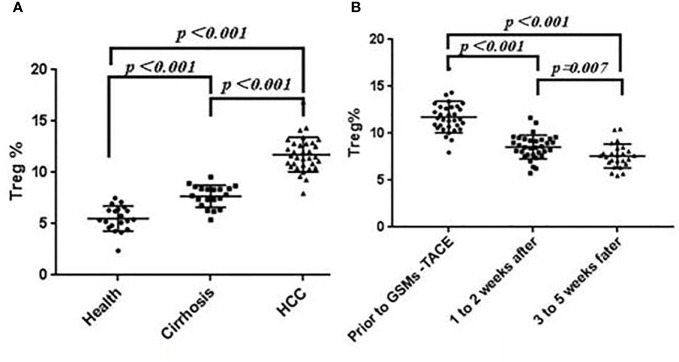
**(A)** Comparison of Treg Cell proportions in Health, Cirrhosis and HCC patients. **(B)** Changes of Treg cell proportions in peripheral blood of HCC patients before and after GSMs-TACE.

The proportion of Treg cells (CD25^+^, CD127+ Low) in Th cells (CD3+, CD4+) was 11.74 ± 1.67% in HCC patients, 5.51 ± 1.22% in healthy group and 7.69 ± 1.07 in Cirrhosis. The proportion of Treg cells in peripheral blood of Cirrhosis and HCC patients was significantly higher than that of healthy group (P < 0.01). See **Figure 2A**.

### The Association Between the Treg Cells Proportion and Clinical Characteristics of HCC Patients

The peripheral blood Treg cell proportion in HCC patients was associated with tumor stage, AFP, PIVKA-II, tumor size, tumor encapsulation, vascular invasion. The proportion of Treg cells in peripheral blood of HCC patients at stage C was higher than that of patients at stage B. The Treg cells proportion in AFP and PIVKA-II positive (≥400 ng/mL; ≥1,000 mAU/ml) HCC patients was higher than that in AFP and PIVKA-II negative patients. In addition, the larger the tumor size (>10cm), the higher the Treg cell proportion in peripheral blood (P < 0.05). According to the enhanced CT examination and Digital Subtraction Angiography(DSA) tumor staining, the Treg proportion in HCC patients without tumor encapsulation or with vascular invasion was higher than that with tumor encapsulation or without vascular invasion (P < 0.01) (**Table 2**). Analysis showed the number of tumor, extrahepatic metastasis, hepatitis B virus-related was not positively correlated with the Treg cell proportion (P>0.05).

**Table 2:** The association between the Treg cell proportion with clinical characteristics of HCC patients.

Characteristics		Number	Treg cells proportion	*t* value	*p*
Tumor stage (BCLC)	stage B	31	9.88 ± 1.89		
	stage C	48	11.49 ± 2.20	3.438	0.001
AFP (ng/mL)	<400	32	10.10 ± 1.77		
	≥400	47	11.37 ± 2.40	2.735	0.008
PIVKA-II(mAU/ml)	<1,000	35	10.08 ± 1.66		
	≥1,000	44	11.47 ± 2.42	2.899	0.005
Tumor size (cm)	<10	41	10.35 ± 1.87		
	≥10	38	11.40 ± 2.45	2.138	0.036
Number of Tumor	<4	43	10.73 ± 1.98		
	≥4	36	11.00 ± 2.50	0.513	0.609
Tumor encapsulation	yes	37	10.12 ± 1.94		
	no	42	11.50 ± 2.27	2.909	0.005
Extrahepatic	yes	33	10.88 ± 2.46		
Metastasis	no	46	10.84 ± 2.05	0.070	0.945
Portal or hepatic	yes	42	11.58 ± 2.28		
Vein invasion	no	37	10.04 ± 1.87	3.294	0.001
Type of hepatitis B	Positive	67	10.87 ± 2.09		
	Negative	12	10.80 ± 2.97	0.075	0.942
HBeAg	Positive	18	10.67 ± 2.04		
	Negative	49	10.94 ± 2.12	0.474	0.638

### The Treg Cell Proportion in Peripheral Blood of 33 HCC Patients Before and After GSMs-TACE

**Figure 3:**
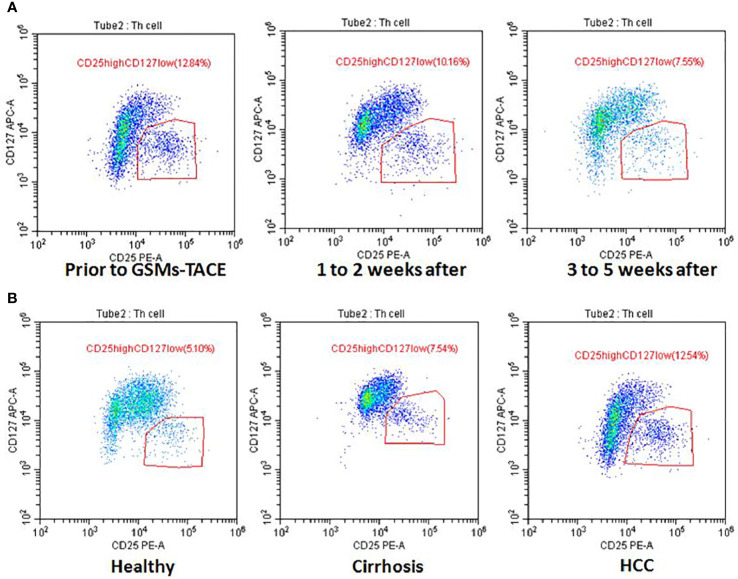
**(A)** Flow Cytometry Chart: The Treg cell proportion of a HCC patient before GSMs-TACE and at 1 to 2 weeks and 3 to 5 weeks after GSMs-TACE was 12.84, 10.16, 7.55%, respectively. **(B)** Flow Cytometry Chart: The Treg cell proportion of healthy people, cirrhosis and HCC patient was 5.10, 7.54, 12.54%, respectively.

In this study, the Treg cell proportion in HCC patients before and at 1 to 2 weeks, 3 to 5 weeks after GSMs-TACE showed a decreasing trend, 11.74 ± 1.67% before GSMs-TACE to 8.54 ± 1.27% at 1 to 2 weeks and 7.59 ± 1.27% at 3 to 5 weeks after GSMs-TACE, which was statistically significantly lower after GSMs-TACE (P < 0.01) (**Table 3**, **Figure 2B**). Representative profiles of high CD25 and low CD127 expressions in the peripheral blood of a HCC patient are shown in **Figure 3A**.

**Table 3:** Comparison of Treg cell proportions in 33 HCC patients before and at 1 to 2 weeks, 3 to 5 weeks after GSMs-TACE.

Time point	No.	Tregs proportion	*t*	*P*
Prior to GSMs -TACE		33	11.74 ± 1.67	
1 to 2 weeks after GSMs -TACE	33	8.54 ± 1.27^#^	8.737	<0.001
3 to 5 weeks after GSMs -TACE	25	7.59 ± 1.27^#^	10.707	<0.001

^#^Treg proportions at 1 to 2 weeks, 3 to 5 weeks after GSMs -TACE were compared with those before GSMs -TACE, P < 0.05.

Moreover, the analysis of the BCLC subgroup showed that the Treg proportion of both stage B and stage C patients decreased significantly at 1 to 2 weeks and 3 to 5 weeks after GSMs-TACE (P < 0.01) (**Table 4**).

**Table 4:** Comparison of Treg Cell proportions in HCC patients with BCLC staging B and staging C HCC before and after GSMs-TACE.

Stage	Time	No.	Treg(%)	*t*	*P*
B	Prior to GSMs -TACE	13	11.16 ± 1.43		
	1 to 2 weeks after	13	8.09 ± 1.10^#^	6.126	<0.001
	3 to 5 weeks after	8	7.07 ± 1.23^#^	16.691	<0.001
C	Prior to GSMs -TACE	20	12.12 ± 1.76		
	1 to 2 weeks after	20	8.83 ± 1.31^#^	6.714	<0.001
	3 to 5 weeks after	17	7.83 ± 1.48^#^	8.630	<0.001

^#^Treg proportions at 1 to 2 weeks and 3 to 5 weeks after GSMs-TACE were both compared with those before GSMs-TACE, P < 0.01.

### The CD4+ CD8+ and NK Cell Proportions in Peripheral Blood of 33 Patients With HCC Before and After GSMs-TACE

**Figure 4:**
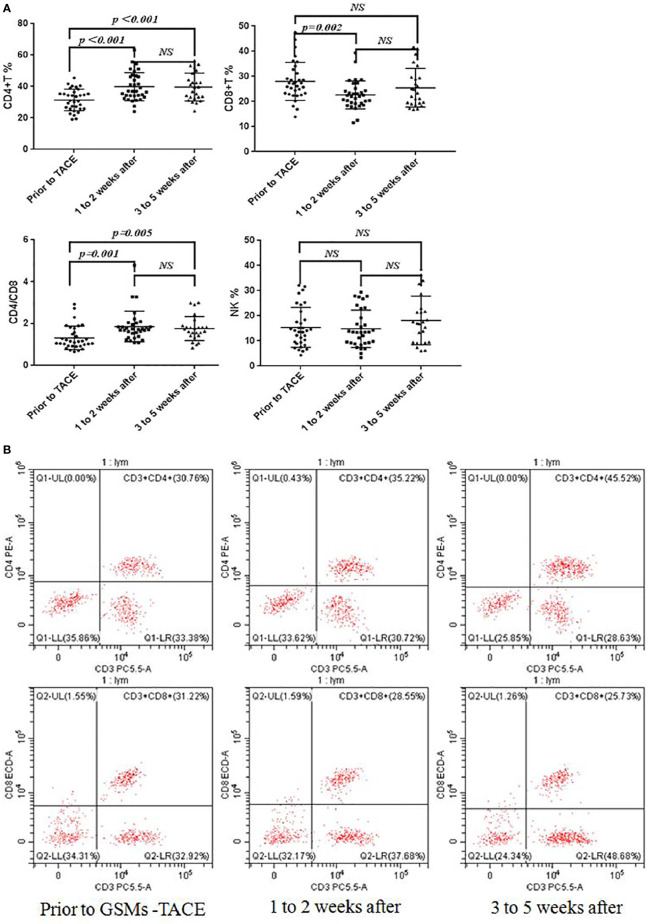
**(A)** Flow Cytometry Chart: Changes of CD4+T, CD8+T, CD4+/CD8+T and NK cell proportions in peripheral blood of HCC patients before and after GSMs-TACE. **(B)** Changes of CD4+T, CD8+T cell proportions in peripheral blood of a representative HCC patient before and after GSMs-TACE detected by flow cytometry.

The comparison between prior to and after GSMs-TACE is demonstrated in **Table 5**, **Figure 4**. The proportion of CD4+T cells of HCC patients was 31.45 ± 6.82, 39.87 ± 8.96, 39.64 ± 8.83% respectively prior to and 1–2 weeks and 3–5weeks after GSMs-TACE (P<0.01), CD8+T cells was 28.01 ± 7.56, 22.64 ± 5.59, 25.47 ± 7.70% respectively. The ratio of CD4+/CD8+T cells was 1.31 ± 0.56, 1.86 ± 0.73, 1.76 ± 0.58% (P<0.01) respectively. These results suggested that the partial T lymphocytes immune function was restored in HCC patients following GSMs-TACE.

**Table 5:** Comparison of CD4+T, CD8+T, CD4+/CD8+T and NK cell proportions in the peripheral blood of HCC patients before GSMs-TACE and at 1 to 2 weeks, 3 to 5 weeks after GSMs-TACE.

Group	Time	HCC patients (n=33)	*t*	*P*
CD4+T (%)	Prior to GSMs -TACE	31.45 ± 6.82		
	1 to 2 weeks after	39.87 ± 8.96	4.294	<0.001
	3 to 5 weeks after	39.64 ± 8.83	3.847	<0.001
CD8+T (%)	Prior to GSMs -TACE	28.01 ± 7.56		
	1 to 2 weeks after	22.64 ± 5.59	3.283	0.002
	3 to 5 weeks after	25.47 ± 7.70	1.256	0.215
CD4+T/CD8+T	Prior to GSMs -TACE	1.31 ± 0.56		
	1 to 2 weeks after	1.86 ± 0.73	3.382	0.001
	3 to 5 weeks after	1.76 ± 0.58	2.939	0.005
NK (%)	Prior to GSMs -TACE	15.38 ± 7.94		
	1 to 2 weeks after	14.85 ± 7.45	0.285	0.777
	3 to 5 weeks after	18.14 ± 9.67	1.161	0.252

### Imaging Changes of HCC Patients Before and After GSMs-TACE

**Figure 5:**
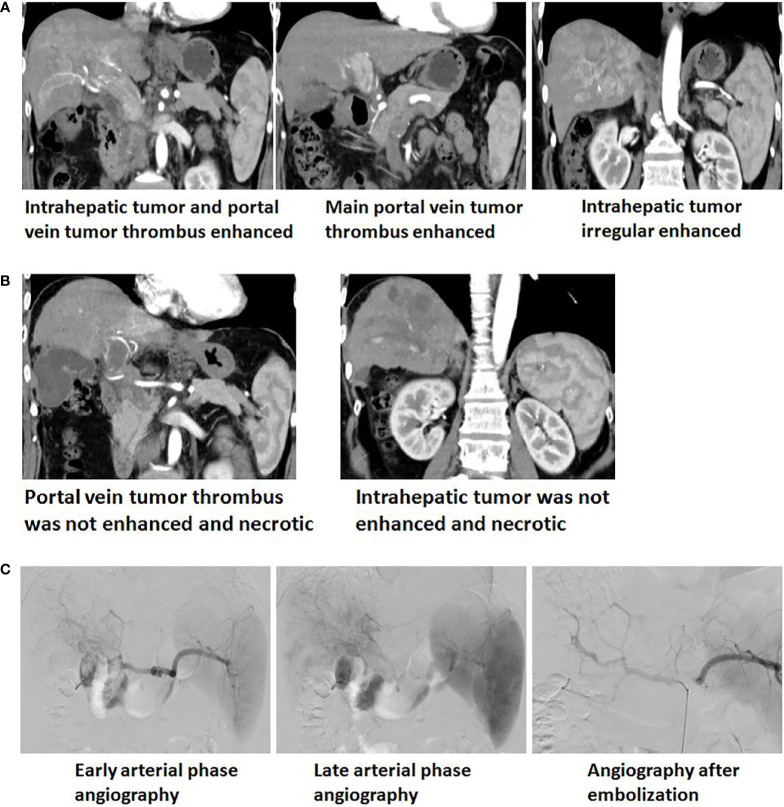
**(A)** Enhanced upper abdominal CT(Arterial phase) showed that the obviously enhanced of irregular tumor and tumor thrombi in the left and right branches of portal vein, which extending to the main portal vein and the opening of splenic vein. **(B)** One month after GSMs-TACE, enhanced CT of the upper abdomen showed that reduction and necrosis of intrahepatic tumor and portal vein tumor thrombus. **(C)** Early and late arterial phase angiography showed arterial staining of intrahepatic tumor and tumor thrombi of portal vein and no distinct boundary with normal liver tissue. Angiography after embolization showed staining of intrahepatic tumor and thrombi disappeared and local arterial blood stasis.

DSA imaging during GSMs-TACE procedures showed intrahepatic tumor staining, and the feeding artery came from the right hepatic artery. After GSMs-TACE, the blood supply artery of the tumor was blocked, and the tumor staining disappeared according to the angiography. On the 4th day after interventional therapy, CT scan of the upper abdomen of 33 patients with HCC showed honeycomb necrosis to different degrees (**Figure 5**).

## Discussion

Currently, HCC is still one of the primary causes of cancer deaths, although the survival rates and prognosis of HCC have been greatly improved by liver transplantation and surgical resection (2). TACE procedures are currently widely used for various cases of HCC, mostly patients at BCLC stage B. Gelatin sponge microparticle (GSM) is an effective embolization agent, and a study reported that when compared with the ethiodized oil which is a permanent embolization agent, GSMs had many advantages, such as a higher tumor response rate at 1 month after treatment, especially in large tumors (13). Our previous clinical practices have confirmed the safety and efficacy of GSMs-TACE (5, 14, 15). It was found that 350–560 μm GSMs combined with single chemotherapy drug TACE procedures was effective and safe in treating elderly HCC without surgical resection, and more elderly patients may have better prognosis (5). GSMs combined with trans-arterial p53-gene-embolization also achieved satisfactory efficacy in treating BCLC stage B HCC as evidenced by favorable survival rates (100% for both 6 and 12 months) and no significant complications (14). Therefore, GSMs were adopted in this study, and imaging scan confirmed the efficacy of GSMs-TACE. DSA angiography during the procedure revealed that after GSMs embolization, the blood flow velocity of tumor feeding arteries, slowed down, and the tumor staining basically disappeared. Moreover, 4 days after GSMs-TACE, CT plain scan showed some honeycomb-like low-density changes in tumors compared with before the procedure. In addition, in a previous study, after analyzing the data of 37 patients who received GSMs-TACE for liver metastases after gastrointestinal tumor surgery, it was observed that the treatment produced various degrees of necrosis and shrinkage of lesions, and even 2 patients achieved a complete response (16). Considering the close relationship between tumor metastasis and the immunosuppressive TME, these findings prompted us to wonder whether tumor necrosis after GSMs-TACE improved the therapeutic efficacy *via* regulating the immune-mediated elimination of tumor cells.

Regulatory T (Treg) cells are a subset of CD4^+^ T cells in immune system known to function as “immune-suppressor”. It is well recognized that Treg cells play an important role in protecting against excessive inflammation and immune response in physiological or some pathological conditions. Furthermore, it has been noticed that Treg cells are involved in liver diseases including HCC (17). The infiltration of Treg cells into tumor sites can exert a suppressive effect on host anticancer immunity and thereby become an obstacle to curative anticancer therapy (10, 18, 19). In the current study, we first compared the Treg cell proportion in peripheral blood in HCC patients, healthy volunteers and Cirrhosis (11.74 ± 1.67%, vs. 5.51 ± 1.22%, vs. 7.69 ± 1.07%, p<0.01), which was in accordance with the previous study (19). Besides, we analyzed the association between the Treg cell proportion and various clinical indicators of HCC, and it was found that the Treg cell proportion was closely related to tumor stage, tumor markers:AFP and PIVKA-II, tumor diameter, tumor encapsulation and vascular invasion, which further confirmed the association between the Treg cell proportion and HCC. However, the mechanism of the association between circulating Treg cells and HCC pathophysiological characteristics has not been fully elucidated. Shi et al. proposed that Tregs could modify HCC in the way that potentiates the metastasis since it was found that Treg cell proportions in HCC patients were significantly correlated with biomarkers of tumor cell metastasis such as E-cadherin, vimentin and SNAIL, although these findings were based on the analysis of limited cases (20). In addition, tumor encapsulation was an important clinicopathological invasive marker of HCC, and it was observed that the high Treg cell proportion in HCC patients was significantly correlated with the presence of tumor encapsulation, which was inconsistent with the published literature, where Gao et al. reported that a high Tregs proportion was associated with the absence of tumor encapsulation (21). Nevertheless, these await further studies.

The 33 patients enrolled in this study received GSMs-TACE, and the imaging techniques confirmed the success of the procedure. To testify our hypothesis that TACE procedures may also have an impact on anti-cancer immunity, the Treg cell proportion in peripheral blood was determined before and at 1 to 2 weeks and 3 to 5 weeks after GSMs-TACE. It was found that circulating Treg cell after TACE was significantly lower than that of before the TACE. These results indicated that GSMs-TACE may have a positive regulatory effect on immune function. The possible reason is that after the effective embolization of tumor feeding artery, the tumor cell necrosis is induced significantly in a short time, and the tumor load is also significantly reduced, which is related to the significant reduction of the immunosuppressive effect on the body. It is well recognized that many tumors bear tumor antigens and can induce T-cell cytotoxic responses (22). The release of tumor antigens then led to local microenvironment immune response and a subsequent reduction of Treg cells. We considered that the “reduction of Tregs” seen in these studies was a relative reduction in frequency reflecting the heightened generation and effectors potentially stimulated by immune-stimulating damage to the tumor itself. The comparison between prior to and after GSMs-TACE, the ratio of CD4+/CD8+T cells increased in HCC patients. These results suggested that the partial T lymphocytes cellular immune function was restored in HCC patients following GSMs-TACE. Our results showed that the improvement of immune function can be maintained within at least one month, which indicated that it may be the optimal time for immunoadjuvant therapy. Therefore, the follow-up treatment including TACE, targeted therapy and immunotherapy can further reduce the burden of tumors in order to better restore and enhance the body’s anti-tumor immunity.

In recent years, tumor immunotherapies have gained promising results. Programmed cell death protein 1 (PD-1), expressed by various activated immune cells, is an immune checkpoint and can protect against autoimmune responses. It has been revealed that in cancer immunity, tumor cells express PD-L1, the ligand of PD-1, and combine with PD-1 in T cells, thereby suppressing T cell activities and promoting the differentiation of Treg cells (23). As a result, the anticancer immune responses are inhibited in tumor microenvironment. The immunotherapies targeting PD-1/PD-L1 have successfully improved the outcomes of various types of cancer worldwide, including HCC (24). A pioneering study analyzed the PD-L1 expression in 240 HCC patients who received surgical resection, and it was found that patients who were positive for PD-L1 showed significant shorter disease-free survival or overall survival than those PD-L1 negative patients (25). Antibodies of PD-1 or PD-L1 in HCC immunology have shown promising efficacies (26). However, there are still unmet clinical needs considering that many patients show no response. Since one of the important mechanisms of PD-1/PD-L1 pathway is the promotion of Treg cells differentiation, an interesting and promising suggestion was drawn from this study that GSMs-TACE has the potential to be used in combination with immune adjuvant therapies such as PD-1/PD-L1 pathway targeting therapies to increase the efficacy of HCC treatment.

In conclusion, the Treg cell proportion of HCC patients was higher than that of healthy and cirrhosis controls and was closely related to the clinical characteristics of HCC. The GSMs-TACE procedure significantly reduced the peripheral blood Treg cell proportions at 1 to 2 weeks and 3 to 5 weeks days after TACE. These results indicated that GSMs-TACE could exert a positive regulatory effect on the Treg, CD4+, CD8+T cell immune function of HCC patients. This study provides a piece of preliminary evidence that GSMs-TACE has the potential to be used in combination with immune adjuvant therapies such as therapies targeting PD-1 or PDL-1 to increase the efficacy of HCC treatment.

However, there are also some limitations of this study, the subgroups of nTreg, iTreg, and tTreg were not detected in detail. Next we’re going to expand our research to supplement data on the activation status of Tregs and the expression of its markers (such as tumor invasion potential, inhibitory medium level). The expression of other molecules or biomarkers involved in tumor immunity should be assessed along with the Treg cell proportion to better explain the mechanism of GSMs-TACE procedures affecting tumor immunity, so as to provide references for the treatment design of HCC patients.

## Data Availability Statement

The original contributions presented in the study are included in the article/supplementary material. Further inquiries can be directed to the corresponding authors.

## Ethics Statement

The studies involving human participants were reviewed and approved by Tsinghua University Affiliated Beijing Tsinghua Changgung Hospital ethics committee. The patients/participants provided their written informed consent to participate in this study. Written informed consent was obtained from the individual(s) for the publication of any potentially identifiable images or data included in this article.

## Author Contributions

First author: ZR. Co-first author: YY. Corresponding author: YWZ. Co-corresponding author: JD. All authors, contributed to the article and approved the submitted version.

## Funding

This work was supported by National Natural Science Foundation of China (81930119), Grant from the Tsinghua Precision Medicine Foundation (12020B7028).

## Conflict of Interest

The authors declare that the research was conducted in the absence of any commercial or financial relationships that could be construed as a potential conflict of interest.
